# Tumor‐stroma ratio is associated with Miller‐Payne score and pathological response to neoadjuvant chemotherapy in HER2‐negative early breast cancer

**DOI:** 10.1002/ijc.33700

**Published:** 2021-06-05

**Authors:** Sophie C. Hagenaars, Stefanie de Groot, Danielle Cohen, Tim J. A. Dekker, Ayoub Charehbili, Elma Meershoek‐Klein Kranenbarg, Marjolijn Duijm‐de Carpentier, Hanno Pijl, Hein Putter, Rob A. E. M. Tollenaar, Judith R. Kroep, Wilma E. Mesker, Dutch Breast Cancer Research Group (BOOG)

**Affiliations:** ^1^ Department of Surgery Leiden University Medical Center Leiden The Netherlands; ^2^ Department of Medical Oncology Leiden University Medical Center Leiden The Netherlands; ^3^ Department of Pathology Leiden University Medical Center Leiden The Netherlands; ^4^ Department of Endocrinology Leiden University Medical Center Leiden The Netherlands; ^5^ Department of Medical Statistics and Bioinformatics Leiden University Medical Center Leiden The Netherlands

**Keywords:** breast cancer, Miller‐Payne, neoadjuvant, pathological response, tumor‐stroma ratio

## Abstract

The tumor‐stroma ratio (TSR) has proven to be a strong prognostic factor in breast cancer, demonstrating better survival for patients with stroma‐low tumors. Since the role of the TSR as a predictive marker for neoadjuvant chemotherapy outcome is yet unknown, this association was evaluated for HER2‐negative breast cancer in the prospective DIRECT and NEOZOTAC trials. The TSR was assessed on 375 hematoxylin and eosin‐stained sections of pre‐treatment biopsies. Associations between the TSR and chemotherapy response according to the Miller‐Payne (MP) grading system, and between the TSR and pathological response were examined using Pearson's chi‐square, Cochran‐Armitage test for trend and regression analyses. A stroma‐low tumor prior to neoadjuvant chemotherapy was significantly associated with a higher MP score (*P* = .005). This relationship remained significant in the estrogen receptor (ER)‐negative subgroup (*P* = .047). The univariable odds ratio (OR) of a stroma‐low tumor on pathological complete response (pCR) was 2.46 (95% CI 1.34‐4.51, *P* = .004), which attenuated to 1.90 (95% CI 0.85‐4.25, *P* = .119) after adjustment for relevant prognostic factors. Subgroup analyses revealed an OR of 5.91 in univariable analyses for ER‐negativity (95% CI 1.19‐29.48, *P* = .030) and 1.48 for ER‐positivity (95% CI 0.73‐3.01, *P* = .281). In conclusion, a low amount of stroma on pre‐treatment biopsies is associated with a higher MP score and pCR rate. Therefore, the TSR is a promising biomarker in predicting neoadjuvant treatment outcome. Incorporating this parameter in routine pathological diagnostics could be worthwhile to prevent overtreatment and undertreatment.

**Abbreviations**
AC‐T: doxorubicin/cyclophosphamide followed by docetaxel
CI: confidence interval
ER: estrogen receptor
FEC‐T: fluorouracil/epirubicin/cyclophosphamide followed by docetaxel
H&E: hematoxylin and eosin
HER2: human epidermal growth factor receptor 2
K: kappa
MP: Miller‐Payne
OR: odds ratio
pCR: pathological complete response
SD: standard deviation
TAC: docetaxel/doxorubicin/cyclophosphamide
TNBC: triple negative breast cancer
TSR: tumor‐stroma ratio

**What's new?**

Neoadjuvant chemotherapy is increasingly used in the treatment of early‐stage, operable breast cancer. However, response rates vary widely among patients, calling for predictive biomarkers. Here, the authors show that a low amount of stroma in pre‐treatment biopsies is significantly associated with a higher chemotherapy response, as measured with the Miller‐Payne grading system and a higher complete pathological response rate in HER2‐negative breast cancer patients. The results highlight the tumor‐stroma ratio, which can easily be incorporated into routine pathological diagnostics, as a promising predictive biomarker for neoadjuvant treatment outcome in breast cancer patients.

## 1 INTRODUCTION

Breast cancer is the leading type of cancer among women in Europe.^1^ Specifically for early‐stage, operable breast cancer, neoadjuvant chemotherapy is increasingly used to induce downsizing of the tumor in the breast and axilla, to increase the rate of breast‐conserving therapy and decrease local therapy of the axilla.^2^, ^3^, ^4^ However, response rates vary immensely among patients who are treated with this treatment modality, mainly depending on their tumor subtypes.^5^, ^6^ This heterogeneity between breast cancer patients has led to an increased interest in predictive biomarkers, as these could improve clinical decision making in early‐stage breast cancer.^7^

The tumor‐microenvironment holds opportunities in the search for a marker to predict treatment efficacy.^8^ Tumor‐associated stromal cells have already been recognized as a prognostic parameter^9^, ^10^ and also show great potential as a possible predictor of neoadjuvant treatment outcome for several cancer types.^11^, ^12^, ^13^ A prognostic tool to assess this compartment, is the tumor‐stroma ratio (TSR). This parameter has first proven to be a strong prognostic factor for survival when assessed on primary colorectal tumors^14^ and it has since been validated for breast cancer, showing that patients with a high stromal content have a relatively worse prognosis compared to the stroma‐low group.^15^, ^16^, ^17^, ^18^, ^19^, ^20^, ^21^ Moreover, the TSR has shown to be a strong independent prognostic parameter with regard to disease‐free survival and overall survival for human epidermal growth factor receptor 2 (HER2)‐negative tumors.^22^ The TSR is a simple and reproducible biomarker, which can easily be incorporated into routine pathological diagnostics and can be performed without additional costs.^13^

In order to further elucidate the predictive value of this parameter, in this study, the TSR was evaluated on pre‐treatment biopsies of the tumor, together with both the Miller‐Payne (MP) grading system and the pathological complete response (pCR) rate after neoadjuvant chemotherapy on surgical resection material. Hereby, we aimed to predict response to treatment in HER2‐negative, early‐stage breast cancer patients treated in the Dutch Breast Cancer Research Group (BOOG) phase III, randomized, multicenter DIRECT^23^ and NEOZOTAC trials.^24^

## 2 METHODS

### 2.1 Study population

This study analyzed patients who were included in two prospective, randomized, multicenter trials that were coordinated by the Dutch Breast Cancer Research Group (BOOG). Both databases comprised of clinical data of women with a histologically confirmed diagnosis of HER2‐negative, stage II/III early breast cancer who received neoadjuvant chemotherapy. All therapies consisted of antracycline and taxane containing chemotherapy agents. In the DIRECT trial, patients received either doxorubicin/cyclophosphamide followed by docetaxel (AC‐T) or fluorouracil/epirubicin/cyclophosphamide followed by docetaxel (FEC‐T), according to hospital guidelines. Moreover, the patients were randomly allocated to either a fasting mimicking diet 3 days before and on the day of chemotherapy or to a regular diet. In the NEOZOTAC trial, patients were randomly assigned to receive either docetaxel/doxorubicin/cyclophosphamide (TAC) or TAC combined with zoledronic acid.

Detailed study design and inclusion and exclusion criteria of both studies have previously been reported.^23^, ^24^ In short, from February 2014 to January 2018, 131 patients participated in the DIRECT trial (NCT02126449), of whom 129 were eligible (one patient was excluded due to metastases and one patient withdrew informed consent). The NEOZOTAC trial (NCT01099436) included a total of 250 women between July 2010 and April 2012, of whom 246 were evaluated in the final analysis (two patients were ineligible and two withdrew informed consent). Informed consent was obtained from all patients.

Both studies were conducted in accordance with the Declaration of Helsinki and were approved by the Ethical Committee of the Leiden University Medical Center in agreement with the Dutch law for medical research involving human subjects.

### 2.2 Tumor‐stroma ratio

**FIGURE 1:**
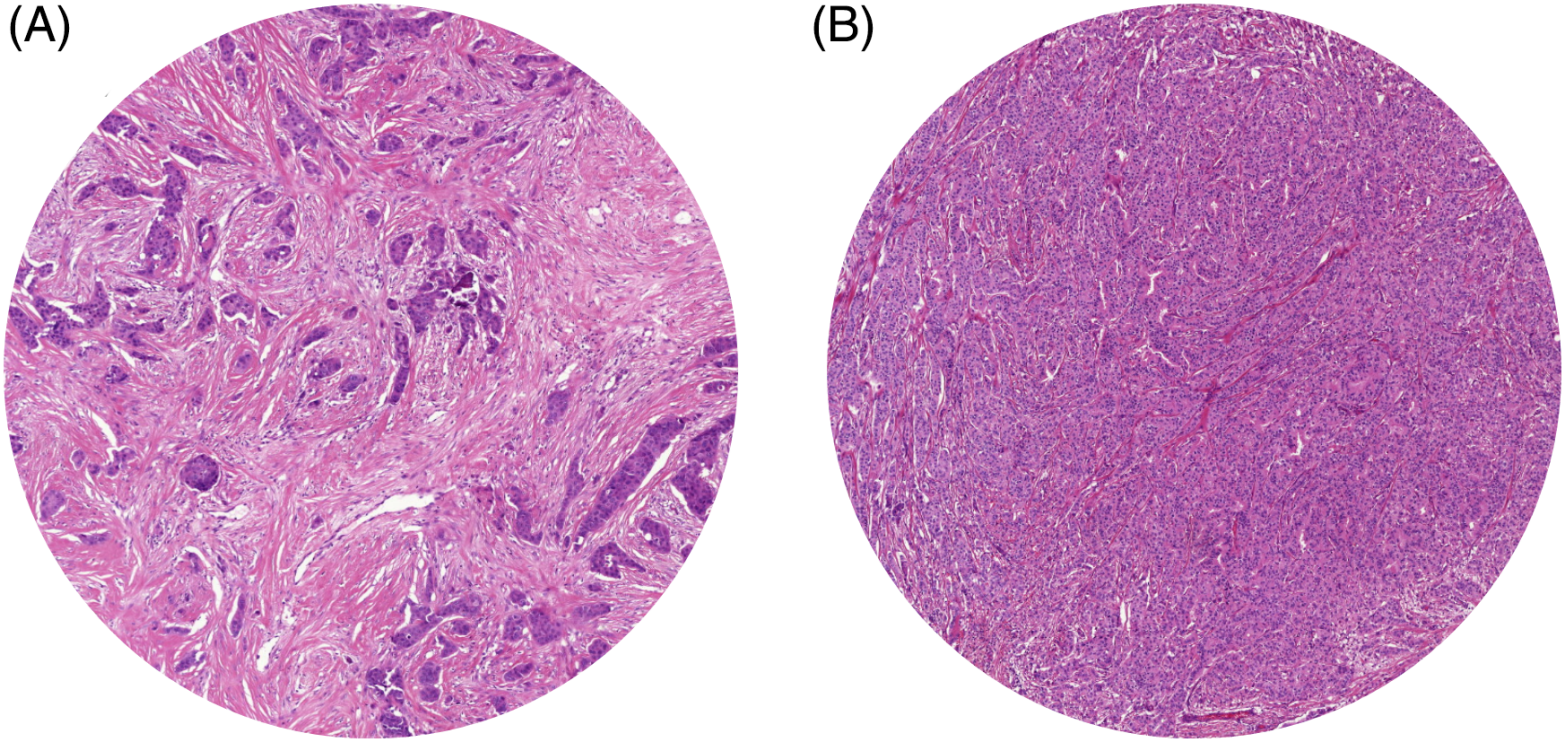
Representative H&E slides of breast tumor tissue for tumor‐stroma ratio evaluation, showing a stroma‐high (A) and a stroma‐low (B) tumor (magnification ×100) [Color figure can be viewed at wileyonlinelibrary.com]

The TSR was visually determined on routine histological hematoxylin and eosin (H&E)‐stained slides of pre‐treatment biopsies, as described previously.^25^ Briefly, only areas were scored where both stromal and tumor cells were present on all four sides of the microscopic field and the TSR was evaluated per tenfold percentage (10%, 20%, etc.). In case of heterogeneity, the highest amount of stroma was deemed conclusive. TSR assessment was performed by a trained researcher and an experienced pathologist. Previous research has indicated an intra‐tumoral stroma percentage of 50% to be a valid cut‐off point.^14^, ^17^ Therefore, a stroma percentage ≤50% correlates to a low amount of stroma (ie, TSR high) and a stroma percentage >50% to a high amount of stroma (ie, TSR low). Tumors were classified as either stroma‐high or stroma‐low (Figure 1).

### 2.3 Endpoints

Primary endpoints were response to neoadjuvant chemotherapy according to the MP grading system^26^ and pCR rate criteria. The MP grading system was defined as a five‐point scale to describe the histological response to neoadjuvant chemotherapy in the primary tumor, with grade 1 showing no decrease in overall tumor cellularity and grade 5 depicting the absence of malignant cells. The definition of pCR was the absence of invasive or in situ tumor cells in the breast and axillary lymph nodes after neoadjuvant therapy.

### 2.4 Statistical analysis

Analyses are reported for the total group of patients and stratified by estrogen receptor (ER) status. Continuous, normally distributed variables are shown as mean (SD) and categorical variables are presented as absolute numbers (percentage). Pearson's chi‐square was used to evaluate possible associations between TSR and pCR rate and between TSR and tumor grade, and the Cochran‐Armitage test for trend for TSR and MP response. Logistic regression analysis was used to estimate odds ratios (ORs) and 95% confidence intervals (CIs) for the association between TSR and pCR rate, adjusting for variables which have been reported to be associated with pCR (age, ER status, tumor grade and tumor [T] stage). The amount of missing values was low and these were, therefore, not imputed. Subgroup analyses of the study groups are provided in the Appendix S1. This study was reported according to REMARK guidelines.^27^ A two‐tailed *P*‐value of less than .05 was considered statistically significant. All data was analyzed using IBM SPSS Statistics (version 25.0 for Windows).

## 3 RESULTS

### 3.1 Patient characteristics

**FIGURE 2:**
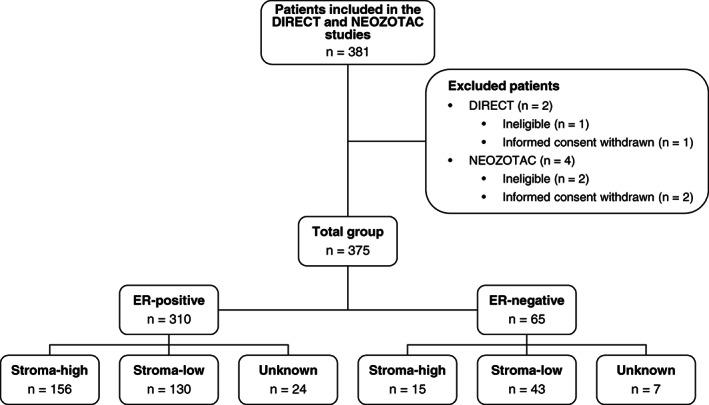
Flowchart of patient inclusion

The total study population comprised of 375 patients with HER2‐negative, stage II/III breast cancer, amenable to neoadjuvant chemotherapy (129 patients as part of the DIRECT trial and 246 patients as part of the NEOZOTAC trial; Figure 2). In the DIRECT trial, 66 (51.6%) tumors were classified as stroma‐low and 62 (48.4%) as stroma‐high. The TSR could not be determined for 1 tumor, because of poor tissue quality. Similarly, in the NEOZOTAC trial, 107 (49.5%) tumors were considered stroma‐low and 109 (50.5%) tumors stroma‐high. The TSR could not be assessed for 30 tumors due to poor quality of the material or missing tissue slides. Mean (SD) age at inclusion was 51.3 (8.4) years and 49.2 (8.0) years for DIRECT and NEOZOTAC, respectively. Distribution of ER status was similar between the groups, with both trials predominantly including patients with ER‐positive tumors (82.9% vs 82.5%). All patient characteristics were well‐balanced between the two trials (DIRECT and NEOZOTAC). A detailed overview of all tumor characteristics is provided in Table 1.

**TABLE 1:** Tumor characteristics of the DIRECT trial and the NEOZOTAC trial

	DIRECT (n = 129)	NEOZOTAC (n = 246)
Mean age (SD), years	51.3 (8.4)	49.2 (8.0)
Stroma status		
High	62 (48.4%)	109 (50.5%)
Low	66 (51.6%)	107 (49.5%)
Unknown	1	30
Clinical T stage		
cT1/cT2	94 (72.9%)	144 (58.5%)
cT3/cT4	35 (27.1%)	102 (41.5%)
Clinical N stage		
cN0	62 (48.1%)	110 (44.7%)
cN+	67 (51.9%)	136 (55.3%)
HR status		
ER−	22 (17.1%)	43 (17.5%)
ER+	107 (82.9%)	203 (82.5%)
Grade (BR)		
I	4 (3.1%)	8 (4.7%)
II	85 (66.4%)	98 (58.0%)
III	39 (30.5%)	63 (37.3%)
Unknown	1	77

Abbreviations: BR, Bloom‐Richardson; ER, estrogen receptor; HR, hormone receptor; N stage, lymph node stage; T stage, tumor stage.

### 3.2 Association between tumor‐stroma ratio and the Miller‐Payne grading system

**FIGURE 3:**
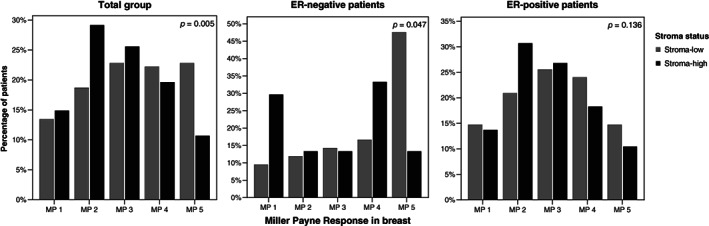
Association between the primary endpoint MP response and the stroma status for the total study cohort and stratified by ER status

Out of 344 patients for whom the TSR was determined, 339 were evaluated for the association between the primary endpoint MP response and the TSR. Five patients were omitted from these analyses, due to incomplete MP response data. Tumors with low stromal content at baseline had a significantly higher MP score, depicting less malignant cells, in the post‐treatment resection specimen (*P* = .005; Figure 3). Subgroup analyses for ER‐negative tumors and ER‐positive tumors showed a similar treatment effect, although this relation was not significant in the ER‐positive group (*P* = .047 and *P* = .136, respectively; Figure 3). Analyses split by trial showed a *P*‐value of .015 for the DIRECT trial subgroup and a *P*‐value of .079 for the NEOZOTAC trial subgroup (Figure S1).

### 3.3 Association between tumor‐stroma ratio and pathological response

**FIGURE 4:**
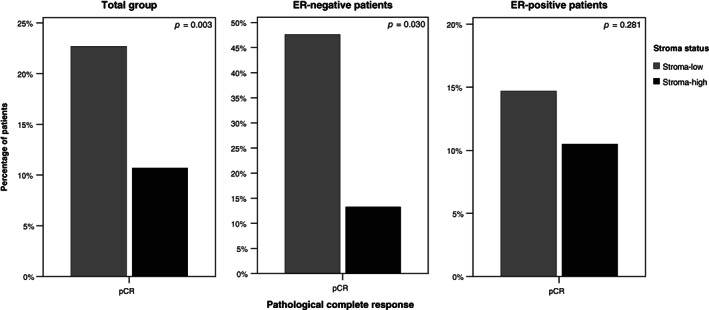
Univariable association between the primary endpoint pCR and the stroma status for the total study cohort and stratified by ER status

Similar to the MP response score, pCR rate was determined for a total of 339 patients, since data regarding pCR was also missing in five cases. pCR was achieved in 16.5% of all women, in 35.9% of the ER‐negative tumors and in 12.4% of the ER‐positive tumors. Pearson's chi‐square revealed a significant association between pCR rate and stroma status for the total group of patients (*P* = .003; Figure 4). In univariable analysis, patients with stroma‐low tumors had a 2.46 higher odds of pCR after neoadjuvant therapy, compared to patients with tumors with a high amount of stroma (OR 2.46, 95% CI 1.34‐4.51, *P* = .004). The ORs and 95% CIs for the multivariable analyses of the pCR rate are shown in Table 2. Multivariable analysis was corrected for age, ER status, tumor grade and T stage. Although no longer significant, there was still a clear trend with a 1.90 higher odds of pCR if patients had a low stromal content (OR 1.90, 95% CI 0.85‐4.25, *P* = .119). In the ER‐negative subgroup (Figure 4), TSR was a statistically significant predictive factor for pCR rate in univariable analysis (OR 5.91, 95% CI 1.19‐29.48, *P* = .030). This was not the case in the ER‐positive subgroup (OR 1.48, 95% CI 0.73‐3.01, *P* = .281). Multivariable analysis could not be performed in the ER‐negative subgroup, due to the undersized patient group. However, in the ER‐positive subgroup, there was a 1.34 odds of pCR if patients had a low stromal content (OR 1.34, 95% CI 0.56‐3.22). Subgroup analyses of the DIRECT and NEOZOTAC trials revealed statistically significant associations between TSR and pCR rate, similar to the total patient group (*P* = .030 and *P* = .030, respectively; Figure S2).

**TABLE 2:** Multivariable odds ratios with 95% confidence intervals for the association between TSR and pCR rate

	Odds ratio	95% confidence interval	*P*‐value
Age	0.954	0.912‐0.998	.040
HR status			
ER−	1		
ER+	0.422	0.182‐0.980	.045
Grade (BR)			
I/II	1		
III	3.040	1.362‐6.784	.007
Clinical T stage			
cT1/cT2	1		
cT3/cT4	0.314	0.127‐0.778	.012
Stroma status			
High	1		
Low	1.898	0.848‐4.250	.119

Abbreviations: BR, Bloom‐Richardson; ER, estrogen receptor; HR, hormone receptor; pCR, pathological complete response; TSR, tumor‐stroma ratio; T stage, tumor stage.

### 3.4 Association between tumor‐stroma ratio and tumor grade

The association between the TSR and tumor grade was evaluated for the total group of patients and the subgroups (Figure S3). Only a few patients (n = 12) were diagnosed with grade 1 tumors. Therefore, the two groups consisting of grade 1 and grade 2 tumors were combined. Pearson's chi‐square showed a statistically significant association between TSR and tumor grade (*P* < .001), with a higher number of grade 3 tumors in the stroma‐low group (73.5%) than in the stroma‐high group (26.5%). This result was also seen in the trial subgroups (*P* = .001 for DIRECT, *P* < .001 for NEOZOTAC).

## 4 DISCUSSION

The aim of this study was to evaluate the predictive value of the TSR on the histopathological response to neoadjuvant anthracycline and taxane‐based chemotherapy in patients with stage II/III HER2‐negative breast cancer. TSR, determined on pre‐treatment biopsies, was equally distributed between the two study groups (DIRECT and NEOZOTAC) and it was predictive for MP response and pCR rate in the total trial population. Low stromal content was significantly associated with a higher MP score and a higher pCR rate in univariable analysis. Similarly, subgroup analysis of ER‐negative tumors revealed a significantly higher MP score and pCR rate for stroma‐low tumors. These outcomes suggest a better response to neoadjuvant treatment for patients with stroma‐low tumors, especially for patients with ER‐negative tumors, compared to patients with stroma‐high tumors.

This study is a planned subanalysis of the two prospective DIRECT and NEOZOTAC trials. In these studies, the effect of dietary interventions on therapeutic efficacy and the addition of zoledronic acid to the neoadjuvant treatment regimen were assessed, respectively. However, both intervention arms did not significantly affect the pCR rate. Therefore, we expect no influence of the interventions on the main objective of the current study.

Importantly, this is the first prospective study investigating the potential of the TSR as a predictor for neoadjuvant chemotherapy outcome in patients with HER2‐negative tumors. A recently published retrospective study analyzing breast cancer biopsies concluded that patients with stroma‐low tumors had a greater chance of good response to neoadjuvant therapy,^28^ similar to our results. Moreover, previous research regarding the value of TSR as a predictor of pathological response in esophageal cancer showed a greater likelihood of nonresponse to neoadjuvant chemoradiotherapy for patients with stroma‐high tumors.^29^ However, our results are in contrast with those presented by Hale et al, who showed a relation between a high proportion of tumor (eg, stroma‐low tumors) and a low amount of tumor regression after neoadjuvant therapy in esophageal carcinoma.^30^ This study estimated the proportion of tumor using a different, semiautomated assessment on digitized pre‐treatment biopsy slides, as opposed to the visual method that is performed by our group.^14^ All previous studies in which the same scoring method was used are thus in accordance with the results presented in this study.

This study does have some limitations. First of all, the TSR was not assessed in lymph nodes in this study, since sentinel lymph node biopsies are currently not standard of care. It was shown that there can be a difference in the stroma status between the primary tumor and the associated lymph nodes and that combining the TSR of the lymph nodes with the TSR of the primary tumor was very valuable in the prognostication of breast tumors.^21^ Thus, assessing the TSR on lymph nodes could be very relevant, considering the heterogeneity in the metastases process between the primary tumor and the lymph nodes. Therefore, further research is required in order to investigate the added value of TSR scoring on lymph node biopsies in the prediction of neoadjuvant treatment outcome.

Secondly, in this study, the assessment of the TSR was performed on pre‐treatment biopsies, instead of on resection material, while the latter is most commonly used in studies. Although the method of scoring the TSR has proven to be easy and reproducible,^13^ the evaluation of biopsies is slightly different and only a smaller part of the tumor is assessed. Courrech Staal et al evaluated the concordance between TSR assessment on primary tumor biopsies and resection material of esophageal adenocarcinomas with the exception of neoadjuvant treated tumors, revealing a Cohen's kappa coefficient (*K*) of 0.506,^25^ while the *K*‐value of TSR scores of breast cancer resection material is higher, ranging from 0.68 to 0.85.^22^ However, the definitive TSR scores of the biopsy and resection material were still the same in 81% of the patients in the esophageal trial. Currently, there is no data available on the concordance between the TSR determined on core‐needle biopsies and resection material of patients with breast cancer.

At present, our hypothesis is that the favorable response to neoadjuvant chemotherapy in the stroma‐low group of our study can be explained as a consequence of tumor biology. It is suggested that stroma provides a favorable, protective environment for the tumor, thereby leading to more aggressive disease, resistance to chemotherapy and worse outcomes in case of stroma‐high tumors.^31^ Thus, tumors with less stroma would be more receptive to chemotherapy. Moreover, previous data from our group showed a possible relationship between the TGF‐β pathway activation and resistance to chemotherapy through misalignment of stromal tissues.^32^ However, the adverse effect of stromal activation is likely governed by multiple pathways. Finally, it has previously been shown in the perioperative chemotherapy (POP) trial that even in patients without systemic therapies, prognosis is relatively favorable in patients whose tumors have low stromal content.^17^ This combination of both a more favorable prognosis and the more pronounced sensitivity to chemotherapy should be taken into consideration when survival outcomes are evaluated in future studies that include the TSR.

In combination with tumor grade and ER status, implementation of the TSR into routine pathological diagnostics could attribute to the prevention of overtreatment of women with breast cancer in the neoadjuvant setting, as well as to the selection of patients that are ideal candidates for such therapies. Results of this study show that patients with stroma‐high, HER2‐negative tumors have a lower rate of pCR after neoadjuvant treatment and have a lower MP response on average, thus less decrease in the amount of tumor cells. Especially for this patient group, considering alternative or additional treatment might be worthwhile. Next to that, the online prediction tool PREDICT can be used in the clinical decision making of early, invasive breast cancer.^33^, ^34^ The addition of the TSR in this model may strengthen its prognostic value and could help with the decision whether to prescribe neoadjuvant treatment to these patients.

Especially within the triple‐negative breast cancer (TNBC) population of this study, patients with a high amount of stroma showed statistically significantly worse outcomes to neoadjuvant treatment. Therefore, personalized therapy would be of great additional value for this group of women, particularly because of the overall worse outcomes that are associated with TNBC.^35^ It could help in defining patients that benefit from additional agents, such as capecitabine, PARP inhibitors and/or immunotherapy. Currently, several studies are ongoing, with the goal of investigating the possibility of administering immune checkpoint inhibitors in addition to neoadjuvant chemotherapy to patients with TNBC. Within both the I‐SPY2 trial^36^ and the KEYNOTE‐522 trial,^37^ a significantly increased pCR rate was seen for patients treated with this regimen in the neoadjuvant setting; a numerical increase in pCR rate was seen in the GeparNuevo study.^38^ Data of the KEYNOTE‐173 trial also suggest that pembrolizumab in addition to neoadjuvant chemotherapy has favorable antitumor activity and a manageable amount of toxicity.^39^ Although preliminary outcomes of the NeoTRIPaPDL1 trial,^40^ in which atezolizumab, carboplatin and *nab*‐paclitaxel are investigated as possible neoadjuvant therapeutic agents in TNBC, did not show similar positive results with respect to pCR rate, the other trials, in which durvalumab (GeParNuevo) and pembrolizumab (I‐SPY2, KEYNOTE‐173 and KEYNOTE‐522) were administered as immunotherapeutic agents in combination with chemotherapy, were very promising and led to an increased pCR rate. These outcomes could possibly be very valuable for the hormone receptor‐negative, stroma‐high patients, who do not respond well to treatment solely consisting of neoadjuvant chemotherapy. Moreover, the TSR could play an additional role in assessing the outcomes in these studies.

In conclusion, within this population of HER2‐negative breast cancer patients who were treated with neoadjuvant chemotherapy, a low amount of stroma in pre‐treatment biopsies of the tumor was associated with a higher MP score and pCR rate. Further research including the stroma status of lymph nodes may enhance the predictive effect of the TSR for treatment outcome to optimize patient selection for neoadjuvant therapy.

## CONFLICT OF INTEREST

The authors declare no potential conflicts of interest.

## ETHICS STATEMENT

Both the DIRECT (NCT02126449) and NEOZOTAC (NCT01099436) studies were conducted in accordance with the Declaration of Helsinki and were approved by the Ethical Committee of the Leiden University Medical Center in agreement with the Dutch law for medical research involving human subjects. Informed consent was obtained from all patients. Trial registration: ClinicalTrials.gov NCT01099436 (NEOZOTAC), registered April 7, 2010 and ClinicalTrials.gov NCT02126449 (DIRECT), registered April 30, 2014.

## Supporting information

**Figure S1** Association between the primary endpoint MP response and the stroma status stratified by study cohort
**Figure S2** Association between the primary endpoint pCR and the stroma status stratified by study cohort
**Figure S3** Association between the tumor grade and the stroma status for the total study cohort and stratified by study cohort

## Data Availability

The datasets analyzed during our study are available from the corresponding author on reasonable request.
