# Supplementary material for: Tumor‐stroma ratio is associated with Miller‐Payne score and pathological response to neoadjuvant chemotherapy in HER2‐negative early breast cancer
Source: Int J Cancer. 2021 Jun 5;149(5):1181–8. doi: 10.1002/ijc.33700 (PMC8362217; doi:10.1002/ijc.33700)
Supplement: Supplementary file 1 — Figure S1 Association between the primary endpoint MP response and the stroma status stratified by study cohort Figure S2 Association between the primary endpoint pCR and the stroma status stratified by study cohort Figure S3 Association between the tumor grade and the stroma status for the total study cohort and stratified by study cohort [file IJC-149-1181-s001.pdf]

Tumor-stroma ratio is associated with Miller-Payne score and pathological response to neoadjuvant chemotherapy in HER2-negative early breast cancer

Sophie C. Hagenaars[#], Stefanie de Groot[#], Danielle Cohen, Tim J.A. Dekker, Ayoub Charehbili, Elma Meershoek-Klein Kranenbarg, Marjolijn Duijm-de Carpentier, Hanno Pijl, Hein Putter, Rob A.E.M. Tollenaar, Judith R. Kroep[*], Wilma E. Mesker[*]; Dutch Breast Cancer Research Group (BOOG).

Table of Contents

Supplementary figure 1 2
Supplementary figure 2 3
Supplementary figure 3 4

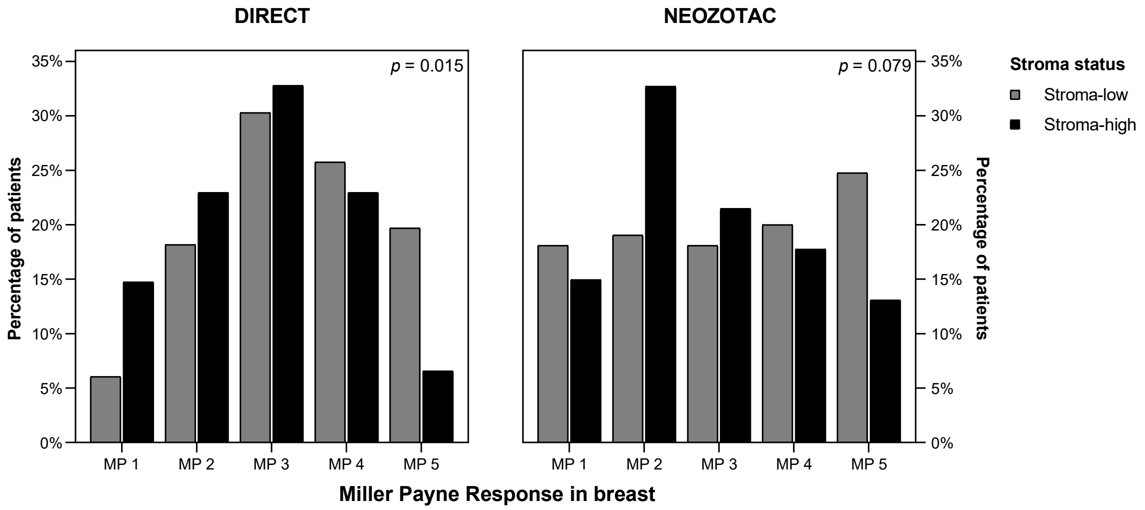

Supplementary figure 1. Association between the primary endpoint MP response and the stroma status stratified by study cohort

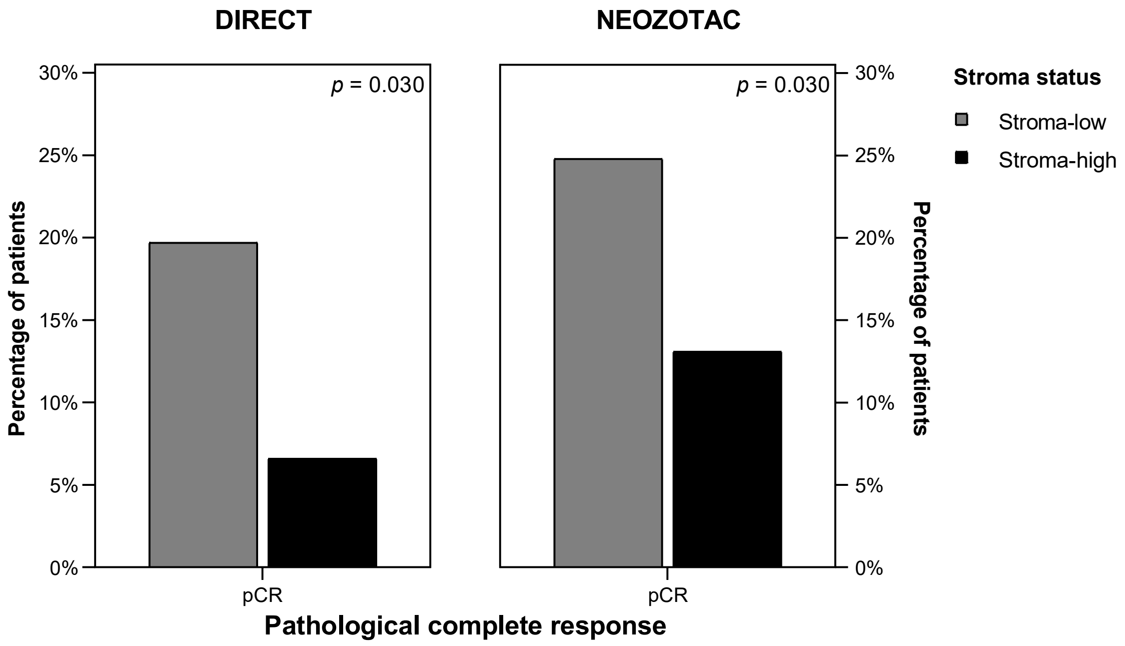

Supplementary figure 2. Association between the primary endpoint pCR and the stroma status stratified by study cohort

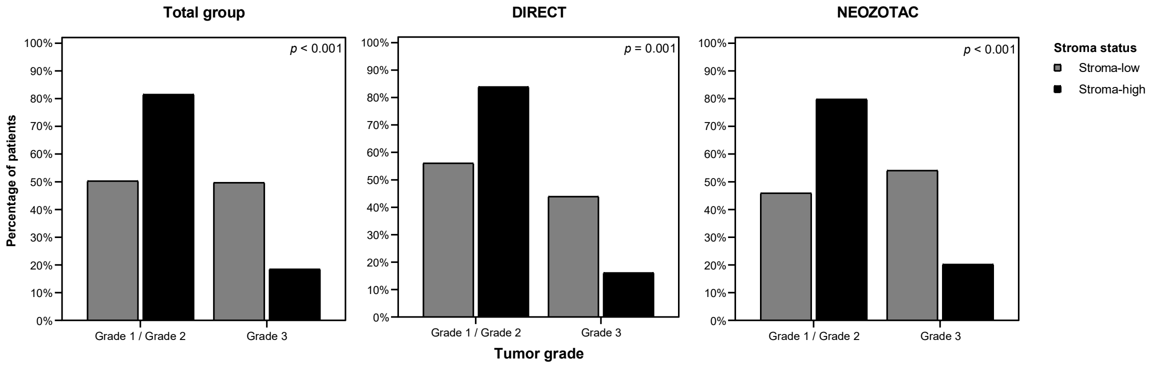

Supplementary figure 3. Association between the tumor grade and the stroma status for the total study cohort and stratified by study cohort
